# APOL1 Dynamics in Diabetic Kidney Disease and Hypertension

**DOI:** 10.3390/biom15020205

**Published:** 2025-02-01

**Authors:** Pravin C. Singhal, Karl Skorecki

**Affiliations:** 1 Department of Medicine, Feinstein Institute for Medical Research, Zucker School of Medicine, Hempstead, NY 11549, USA; 2 Azrieli Faculty of Medicine, Bar-Ilan University, Safed 1311502, Israel; karl.skorecki@biu.ac.il; 3 The Ruth and Bruce Rappaport Faculty of Medicine, Technion—Israel Institute of Technology, Haifa 3525433, Israel; 4 Department of Nephrology, Rambam Health Care Campus, Haifa 3109601, Israel

**Keywords:** APOL1, diabetic kidney disease, chronic kidney disease, hypertension, renin–angiotensin system, miR193a

## Abstract

APOL1 Renal Risk Variants (APOL1RRVs, G1, and G2) are known to be toxic to glomerular podocytes and causally associated with an enhanced prevalence and progression of many different etiologies of chronic kidney disease (CKD), leading to the delineation of a new disease designation of APOL1-Mediated Kidney Disease (AMKD). Notably, APOL1RRVs have not consistently been shown to increase the prevalence or severity of diabetic kidney disease (DKD) progression, which is the most common cause of End-Stage Kidney Disease (ESKD). While this apparent discrepancy seems perplexing, its clarification should provide important mechanistic and therapeutic insights. Activation of the Renin–Angiotensin System (RAS) plays a critical role in the development and progression of DKD. Recent in vitro and in vivo studies also demonstrated that RAS activation contributes to kidney cell injury in AMKD experimental models. Both high glucose, as well as APOL1RRVs escalate the podocyte expression of miR193a, a known mediator of glomerulosclerosis, including idiopathic Focal Segmental Glomerular Sclerosis (FSGS) and DKD. We propose that either the RAS and/or miR193a levels in the diabetic milieu are already maximally conducive to kidney target cell injury and, therefore, are agnostic to further injury in response to APOL1RRVs. Similarly, the contributory role of hypertension (which is frequently reported as the second most common cause of ESKD) in the progression of AMKD remains a controversial issue. Since several clinical reports have shown that controlling hypertension does not consistently slow the progression of AMKD, this has led to a formulation wherein APOL1-RRVs primarily lead to kidney injury with accompanying hypertension. Notably, half a decade later, the notion that hypertension is not a cause but rather a consequence of kidney injury was contested by investigators analyzing the Mount Sinai BioMe repository, a comprehensive clinical and genetic database including participants with APOL1RRVs. These investigators observed that hypertension predated the observed decline in GFR in individuals with APOL1RRVs by ten years. In the present study, we discuss the mechanistic forces that may underpin the gaps in these clinical manifestations, which did not allow the temporal association of hypertension with AMKD to be translated into causation and may also dissociate DKD and AMKD. We have hypothesized models that need to be validated in future experimental studies.

## 1. APOL1 and CKD

Americans with African ancestry (AAAs) have a risk of chronic kidney disease (CKD) that is four times higher than Americans with European ancestry, and a greater risk of family members with kidney disease [1]. This excess burden of CKD cannot be attributed solely to societal and economic factors. One and a half decades ago, variants (G1 and G2) in the apolipoprotein L1 (APOL1; G0) gene were reported with an excess risk of Focal Segmental Sclerosis (FSGS), HIV-Associated Nephropathy (HIVAN), and other forms of CKD and ESKD in AAAs [2,3,4]. The intricate and complex relationship between APOL1 Renal Risk Variants (RRVs, G1 and G2) and CKD presents a fascinating and challenging puzzle. While significantly cytotoxic to podocytes, these variants do not follow the expected pattern in diabetic kidney disease (DKD), a hallmark of diseases associated with loss of podocyte integrity [5]. Disentangling this complexity, which is yet to be fully understood, offers a promising avenue for future research and also holds the potential for significant breakthroughs in understanding kidney disease, inspiring a sense of hope and motivation among investigators.

## 2. What Is APOL1-Mediated Kidney Disease (AMKD)?

In the diseases mentioned above, APOL1 variants seemed to be a stand-alone driver, predominantly proteinuric and podocyte-centric but with a population genetic association with African ancestry [2,3,4]. Notably, collapsing glomerulopathy, which is considered to be a hallmark of APOL1 variant-induced massive podocyte injury in the viral milieu (HIV, COVID-19, and Parvovirus B19), was associated with escalated levels of IFNγ, which provided insight into the mechanistic aspects of these variants’ modus operandi [4,5,6,7,8,9]. Since these diseases carry a common mechanistic driver, linking them as part of the spectrum of APOL1-mediated kidney diseases was logical. The presence of the two variants was consistently demonstrated in many follow-up studies to confer a high renal risk. In contrast, the presence of one variant led to a more minor and, in some studies, imperceptible increase in the risk of CKD with observations extended to continental African populations [10,11,12,13,14,15,16]. Further studies showed that background haplotype variation could confer enhanced risk or protection from APOL1 association with kidney disease, with the emergence of a new category of etiologies of CKD termed APOL1-Mediated Kidney Disease (AMKD) [17,18,19,20,21,22,23].

## 3. APOL1 and Its Variants

APOL1 belongs to a six-member family of APOL genes clustered on chromosome 22 and appeared late in mammalian evolution (≈30–35 million years ago) [24]. It circulates in the blood as a part of an HDL complex and is also expressed by cells, including the lung, placenta, pancreas, liver, and kidney [25,26]. APOL1 encodes a signal peptide, releasing multiple splice isoforms into circulation. The G1 variant comprises two amino acid substitutions, S342G and I384M, and the G2 variant comprises a two-amino-acid deletion (del388N389Y). APOL1 is absent among non-primates and is noted to be present in a few higher-order primates. These variants perhaps evolved 10,000 years ago and became prevalent in Africa because they protected humans from fatal sleeping sickness due to *Trypanosoma brucei* (*Tb*) *rhodesiense*/*gambiense* [27,28]. *Tb rhodesiense* inflicted acute sleeping sickness in East Africa, and *Tb gambiense* caused chronic sleeping sickness in West Africa. APOL1 (G0) conferred protection to humans by lysing Tb species; however, in due course of parasite evolution, *Tb rhodesiense* evolved the serum-resistance-associated (SRA) gene, whose protein product binds directly to the C-terminal domain of the G0 version of APOL1, sequestering and preventing APOL1 from adopting the conformation required to mediate the trypanolytic cascade. *Tb gambiense* utilizes a different mechanism. In turn, the APOL1 G1 variant promotes an asymptomatic carrier state in Tb *gambiense* infection, and the G2 variant protects against *Tb rhodesiense* infection [29,30].

## 4. Do APOL1RRVs Activate Intra-Renal RAS?

**Figure 1:**
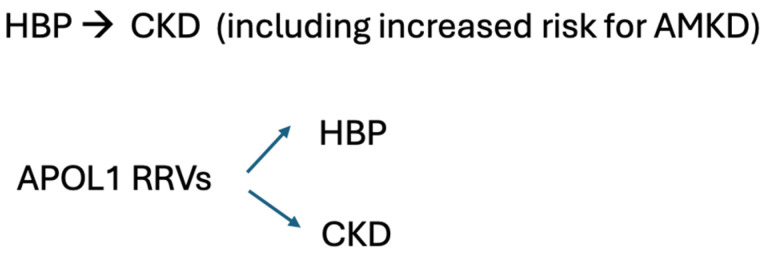
(**Top panel**)—conventional formulation that systemic arterial hypertension causes chronic kidney disease and enhances the risk of progressive AMKD. (**Bottom panel**)—alternative formulation that APOL1 risk variants cause both kidney injury through podocyte toxicity and systemic arterial hypertension as a result of kidney injury or perturbation at other target cells (e.g., endothelial). HBP—high blood pressure. CKD—chronic kidney disease.

Clinical reports indicate that APOL1RRV patients demonstrated a more significant reduction in albuminuria and better blood pressure control if their hypertension was treated with Angiotensin II Receptor Blockers (ARBs) [31]. Additionally, APOL1RRV patients suffering from CKD associated with sickle cell disease showed a more significant reduction in albuminuria when treated with ARBs or Angiotensin I Converting Enzyme (ACE) inhibitors [32]. These reports suggest that intra-renal Renin–Angiotensin System (RAS) activation may contribute to CKD in hypertensive patients carrying APOL1RRV (Figure 1). However, it remains puzzling that diabetes mellitus, the most common cause of CKD [33], and a vibrant example of intra-renal activation of the RAS [34,35], seems not to be affected by APOL1RRVs [1,14,36].

## 5. What Is RAS Status in Diabetic and APOL1 Milieus?

Let us examine the role of the known mechanistic drivers of kidney injury in diabetic and APOL1RRV milieus. We will first address the role of activation of the RAS. In a recent report, treatment with ACE inhibitors in APOL1RRV mice showed a genotype-dependent but striking decrease in proteinuria and significant attenuation of glomerulosclerosis [37], supporting a role for RAS activation in the progression of kidney disease in this humanized murine model. A non-RAS-system antihypertensive agent was ineffective, and, significantly, for the current consideration, a gliflozin class of SGLT2 inhibitors did not ameliorate kidney injury in this model. In in vitro studies, we have also previously demonstrated the activation of the RAS and associated cytotoxicity in podocytes overexpressing APOL1RRVs [38].

## 6. Why Does APOLRRV-Mediated RAS Activation Not Affect the Course of DKD?

**Figure 2:**
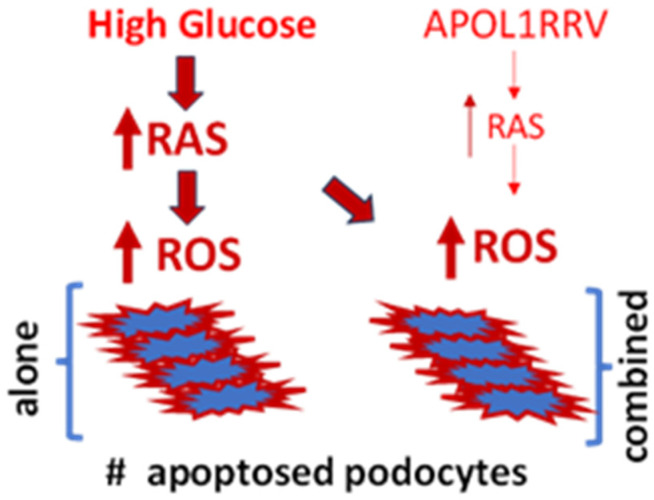
Schema of numbers (#) of apoptotic podocytes in response to high glucose-induced RAS-mediated ROS generation alone vs. in combination with the APOL1RRV milieu. The density and width of the arrows indicate the amount of ROS generation. Renin–Angiotensin System (RAS). Reactive oxygen species (ROS).

We asked why APOL1RRV-mediated RAS activation does not seem to accelerate the course of DKD, and we propose the following potential explanation. Since the diabetic milieu per se is associated with a high level of podocyte RAS activation [39,40,41], the additional mild to moderate level of RAS activation stimulated by APOL1RRVs in diabetic individuals would perhaps not be expected to exacerbate the existing RAS levels any further (Figure 2). This is also evident from the severity of the cytotoxic effect of glucose vs. APOL1RRVs on kidney cells; for example, a high percentage of diabetic patients develop DKD [33], but only 4% of individuals carrying APOL1RRVs develop primary kidney disease-Focal Segmental Sclerosis (FSGS), and that too after a presumed second hit [28]. This indicates the likely involvement of the perturbation of at least one or more additional regulatory axes in the causal association of APOL1RRVs in the context of hypertension and CKD.

## 7. Is miR193a Involved in Masking APOL1RRVs’ Role in DKD?

We propose that one such axis revolves around miR193a, a microRNA that regulates gene expression in podocyte and parietal epithelial cells (PECs) [42]. During embryogenesis, PECs and podocytes originate from the same cellular lineage. In animal experimental mouse models, PECs proliferate and maintain podocyte homeostasis in the juvenile period [43]. Podocyte homeostasis is a crucial process that ensures the normal functioning of the glomerular filtration barrier [44,45]. However, in adult mice, PECs cannot transition to podocytes when exposed to adverse milieus associated with escalated miR193a levels [43]. Since miR193a negatively regulates Wilms Tumor Type (WT) 1, a master transcription factor for podocyte genes, the decrease in miR193a augments podocyte gene expression and optimizes the podocyte molecular phenotype [42]. Because WT1 is also a constituent of the repressor complex, which inhibits transcription of PAX2, a critical PEC gene, the miR193a-mediated attenuated transcription of WT1 would contribute to an optimized PEC molecular phenotype [46]. Thus, in an escalated miR193a milieu, PEC health will be optimized, and they may respond to the loss of podocytes (in adverse milieus associated with enhanced miR193a expression) by proliferation. However, proliferating PECs would not transit to the podocyte phenotype, resulting in a failure in podocyte homeostasis and initiation of glomerulosclerosis.

## 8. Do High Glucose and APOL1RRVs Escalate miR193a Expression?

**Figure 3:**
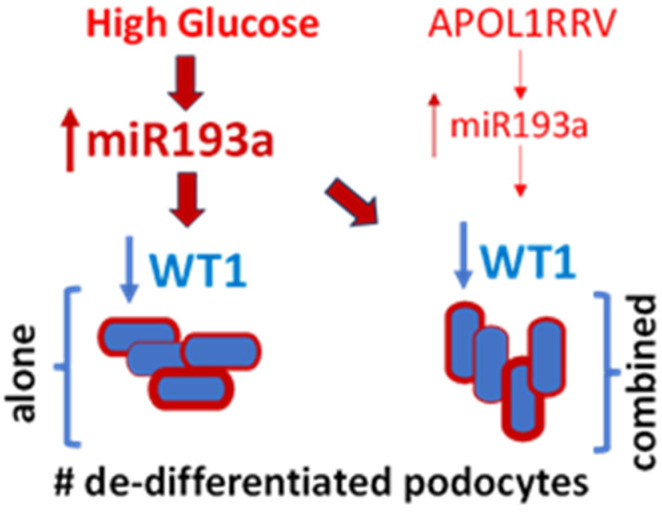
Schema of numbers (#) of dedifferentiated podocytes in response to high glucose-induced miR193a-mediated downing of WT1 expression by podocytes alone vs. in combination with the APOL1RRV milieu. The density and width of the arrows indicate levels of miR193a.

Notably, APOL1 wild-type (APOL1G0) downregulates miR193a, but APOL1RRVs upregulate the expression of miR193a in PECs and podocytes [47]. Since both APOL1RRVs and high glucose escalate the expression of miR193a in podocytes [46,47], enhanced expression of miR193a in podocytes would cause a loss of podocytes, which may invoke PEC proliferation [48]. However, these proliferating PECs would not transit to podocytes because of escalated miR193a levels in an ongoing adverse milieu [49]. It appears that either APOL1RRVs’ stimulating effects were not able to exacerbate high glucose-escalated miR193a levels further or there was a limit for the cytotoxicity of an optimal miR193a level, beyond which it does not increase (Figure 3). It is worth testing these hypotheses in diabetic APOL1RRV-trangenic mice.

## 9. What Are the Effects of miR193a on CKD Progression?

The enhanced expression of miR193a in podocytes is directly linked to the loss of podocytes [50]. This is evidenced by the miR193a transgenic mouse model being an experimental animal model of idiopathic FSGS. Notably, high glucose levels also lead to increased miR193a expression, which has been reported to play a role in developing DKD [46,51,52]. In essence, miR193a plays a significant role in the development of kidney diseases, particularly in the loss of podocytes, and its expression is influenced by factors such as high glucose levels and genetic variants like APOL1RRVs. Understanding the role of miR193a is crucial for advancing our knowledge of kidney disease and developing effective treatments.

## 10. APOL1RRVs and Hypertension

However, another discrepancy revolves around the role of APOL1RRVs in the development of hypertension. The recognition of the role of APOL1-RRVs in the development of CKD was followed by reconsideration of the term hypertensive CKD in patients of African ancestry, with the connotation of hypertension as primarily causative for CKD. Instead, the term hypertension-attributed chronic kidney disease was introduced. It was presumed that these individuals had prior chronic kidney disease related to APOL1RRVs, which contributed to their hypertensive profile. Multiple clinical reports supported this notion [53,54,55]. However, a half-decade later, one study offered a different perspective [56]. The Mount Sinai BioMe repository analysis, a comprehensive clinical and genetic database including participants with APOL1RRVs, demonstrated that individuals aged 20 to 29 years had no decline in eGFR but exhibited an increase in systolic BP of 0.94 mmHg per copy of an APOL1RRV; however, they did show declines in eGFR approximately 10 years later (at the age of 30 to 39 years). This analysis was challenging as it suggested that hypertension predated and might have led to declining eGFR. Based on their findings, those authors asserted that onset of hypertension predates a decline in GFR in the APOL1RRV milieu. The authors further proposed that the earliest effect of *APOL1* might be on hypertension and not on the kidneys, a significant shift in our understanding of the disease.

## 11. BioMe Repository Data Analysis on AMKD Time Course

**Figure 4:**
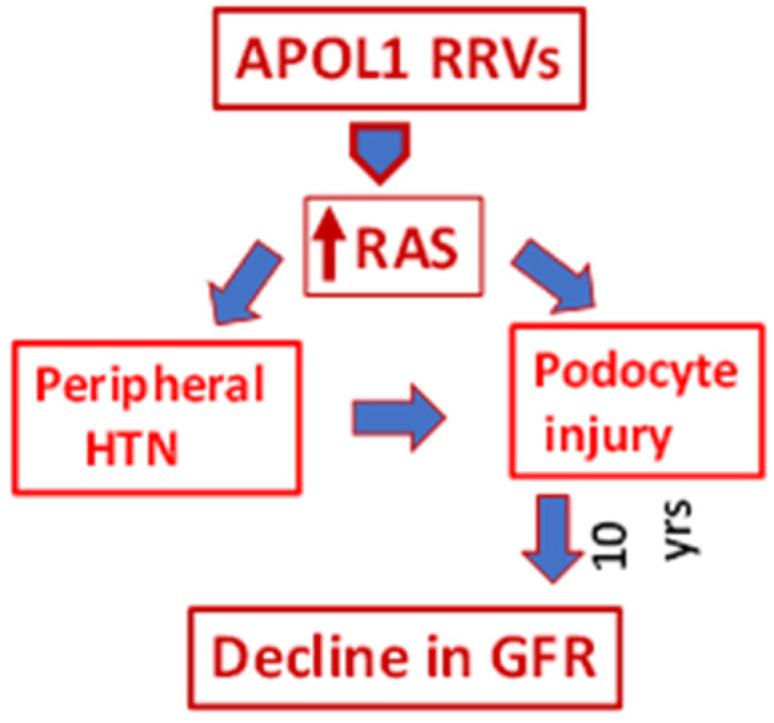
Activation of the Renin–Angiotensin System (RAS) in an escalated APOL1RRV milieu increased peripheral blood pressure and induced podocyte injury of the same type. However, a decline in GFR took 10 years to manifest. Renin–Angiotensin System (RAS).

What do we learn from the BioMe Repository analysis data? Firstly, APOL1RRV directly contributes to the development of hypertension in individuals carrying this genetic profile. Does this mean that hypertension predates kidney disease in the APOL1RRV milieu? Not necessarily. As noted above, recently, the role of the activation of the RAS was highlighted in an animal experimental model of APOL1RRVs [37]. ACE inhibitors attenuated proteinuria in APOL1RRV mice with differential sensitivities. In in vitro studies, APOL1RRV-expressing podocytes displayed enhanced levels of mRNA and protein constituents of the RAS pathway [38]. Moreover, APOL1RRV mice exhibited increased levels of Ang II when compared to control mice. Since only a limited percentage of individuals carrying APOL1RRV develop hypertension or CKD, this suggests that the affected individuals were exposed to the second hit (adverse milieus such as viral infection) for an overt expression APOL1RRVs; the latter contributed to the activation of the RAS and manifested as hypertension systemically but podocyte toxicity in glomeruli; chronologically, during the initial period, systemic hypertension may not affect glomerular capillary pressure because of autoregulation. The net outcome of these events would be an unchanged GFR for several years, masking the structural loss of kidney function (podocyte injury) (Figure 4). These findings open up new possibilities for the initiation of early treatment of systemic hypertension as well as glomerular hypertension or a masked renal injury with RAS blockers in individuals with APOL1RRVs.

## 12. Discrepancy in the Manifestation of Disease Markers in CKD

In the diabetic milieu, increased plasma volume contributes to glomerular hyperfiltration because of hyperglycemia-associated hypervolemia; however, it is also associated with glomerular hypertension (because of loss of autoregulation) and RAS-induced podocyte loss. In time course studies, proteinuria and a decline in GFR begin to manifest approximately 10 years later in type 1 diabetic patients [33]. In type 2 diabetes, the onset of decline for GFR was challenging to ascertain because the onset of diabetes remains undetermined. Since significant factors like high glucose-mediated hypervolemia and glomerular hypertension were missing in the APOL1RRV milieu and RAS-mediated podocyte loss was also milder because of second-hit-triggered APOL1RRV expression levels being variable, a decline in GFR requires a more extended period: approximately 10 years. Intra-renal RAS activation may have remained unidentified at the onset of hypertension in individuals enrolled in the BioMe data bank repository because we lacked the technical capabilities to monitor intra-renal injury, including glomerular hemodynamic status. Urinary podocyte count or other circulating or urinary biomarkers of podocyte injury could elucidate this point. In a nutshell, systemic hypertension is predominantly a hemodynamic event; in contrast, GFR modulation is the net outcome of glomerular cell injury and glomerular hemodynamic perturbations. A net decline in GFR takes time to occur in the APOL1RRV milieu.

## 13. APOL1RRV-Mediated Hypertension vs. Essential Hypertension

Interestingly, APOL1RRV-mediated hypertension differs from essential hypertension because it is also associated with ongoing direct RAS-induced podocyte loss. From that perspective, once there is a loss of autoregulation (after the development of afferent arteriolar sclerosis), APOL1RRV-mediated glomerular damage would be expected to accelerate immensely in APOL1RRV individuals. In that situation, using RAS blockade is expected to be a sound therapeutic strategy, with the caveat of recent provocative research results pointing to the possible engagement of deleterious long-term glomerulosclerosis pathways in a hyper-reninemic setting [57].

## 14. Role of RAS Blockade in AMKD

If intra-renal activation of the RAS had been the main culprit for the progression of CKD, why are we not able to effectively manage it with anti-RAS therapeutic tools? ACE inhibitors, for instance, can only block the RAS up to 50% as the rest of Ang II is generated through the chymase pathway [58]. ARBs, on the other hand, can block the effect of Ang II on AT1R, but they do not decrease the production of Ang II, which also works on AT2R and is known to display other cytotoxic effects. This limitation of the current anti-RAS blockade is often observed during the management of diabetic kidney disease, as well as APOL1RRV-associated kidney diseases. The implications of these findings on the management of APOL1RRV-related kidney disease are significant, guiding future research and clinical practice in this area.

## 15. How Do APOL1 Variants Interact with the RAS, and How Does It Affect CKD Progression?

**Figure 5:**
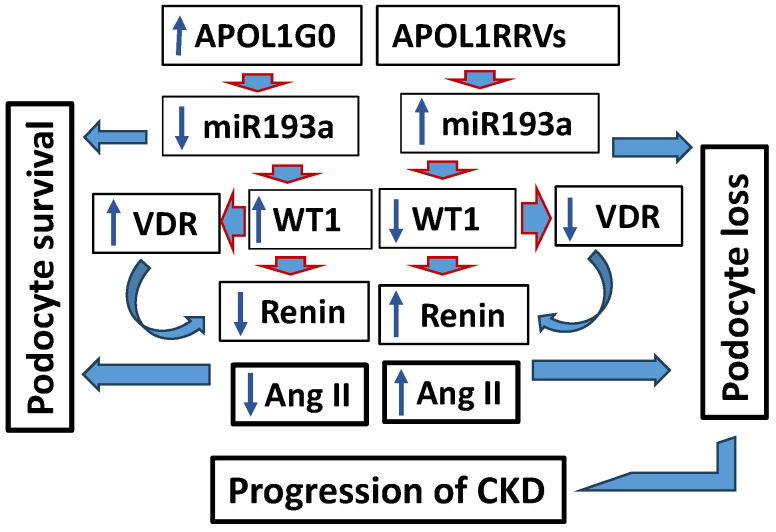
Schematic diagram displaying APOL1RRV-mediated downstream signaling pathway leading the activation of the RAS and participating in the progression of CKD. Vitamin D Receptor (VDR); Wilms Tumor Type (WT) 1.

APOL1G0 negatively regulates miR193a expression in podocytes [46], but the APOL1–miR193a axis is disabled in the context of APOL1 risk variants; conversely, APOL1 variants enhance the expression of miR193a [47], which is a driver of podocyte loss through the inhibition of WT1 [50] and activation of the RAS by attenuating the expression of VDR [38]; the latter negatively regulates the generation of renin [59,60]. Loss of podocytes causes a decrease in GFR and proteinuria, thus initiating tubular interstitial fibrosis and its further progression (Figure 5) [61,62]. Decreased GFR, proteinuria, and the progression of tubular intestinal fibrosis are hallmarks of CKD. Since glomerular hypertension is mediated predominantly through the activation of the local RAS, its downregulation significantly reduces proteinuria. On that account, strategies to slow the progression of CKD, particularly those that aim to decrease proteinuria, prominently include RAS blockade.

## 16. Therapeutic Strategies for DKD and Hypertension in the APOL1 Variant Milieu

Since classical CKD-FSGS occurs in only 4% of individuals carrying APOL1 variants, a theory was proposed that a second hit or exposure to an adverse milieu is required to escalate the expression of APOL1 variants for an optimal time and/or create a hostile environment that is adverse to podocyte health [28]. From that perspective, if individuals carrying APOL1 variants develop podocyte injury because of a second hit, they may then develop CKD. Peripheral hypertension may predate the development of CKD. However, the development of diabetes later in life is dependent on the metabolic phenotype; if that situation persists, the factors associated with these phenotypes will accelerate the pre-existing CKD, which was initiated through escalation of APOL1 variant expression.

**Figure 6:**
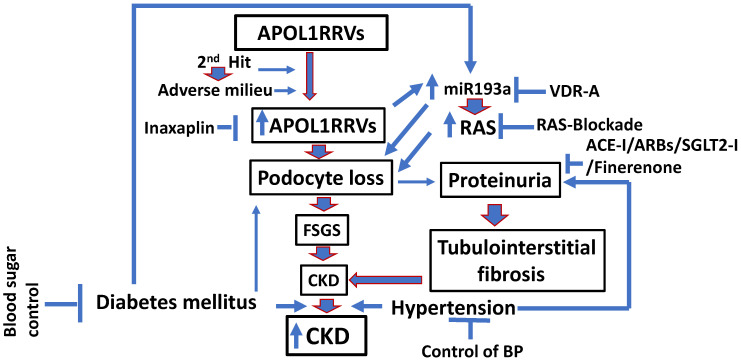
Schematic diagram showing therapeutic strategies for the management of DKD and hypertension in APOL1RVV milieu. Vitamin D Receptor-Agonist (VDR-A); Renin–Angiotensin System (RAS); Focal Segmental Sclerosis (FSGS); Blood Pressure (BP); Angiotensin Converting Enzyme- Inhibitor (ACE-I); Angiotensin Receptor Blockers (ARBs); Sodium–Glucose Cotransporter-2 Inhibitor (SGLT2-I).

As shown in the schema, escalated APOL1RRVs enhance podocyte generation of miR193a, which causes podocyte injury directly through the inhibition of WT1 and indirectly through RAS activation (Figure 6). In this scenario, one may directly inhibit the action of APOL1RRVs by using inaxaplin or its downstream effect on miR193a expression via a VDR agonist [47]. Additionally, the RAS activation could be blocked by vitamin D, ACE inhibitors, and ARBs [59,63]. In real-life situations, patients often approach health care providers after proteinuria-significant loss of podocytes has already occurred; furthermore, the patient has already developed a significant degree of tubulointerstitial fibrosis at this stage. In a nutshell, proteinuria is the result of a combination of intertwining pathophysiologic processes, including a breach of the glomerular filtration barrier (podocyte injury leading to the derangement of slit diaphragm/loss of podocytes), glomerular hypertension (hyperfiltration caused by RAS activation), and attenuated tubular absorption of protein (tubulointerstitial fibrosis, initiated by proteinuria). Therefore, the current strategies attempt to slow progression rather than prevent CKD. Under normal physiological conditions, systemic hypertension does not transmit to afferent arterioles because of the existing autoregulation. However, in the case of diabetes or advanced CKD, autoregulation is lost, and systemic blood pressure is transmitted to glomerular capillaries, causing escalation of glomerular pressure, which not only damages glomeruli but also increases proteinuria. Since proteinuria is a key marker of the progression of CKD [60,61] and is also a risk factor for stroke and cardiac events, controlling hypertension should be one of the central pillars of managing CKD in APOL1RRV individuals suffering from either DM, hypertension, or both. Additional agents that are often used to decrease proteinuria include SGLT2 inhibitors and synthetic and mineralocorticoid antagonists (e.g., finerenone), which predominantly work on tubular health [64,65,66]; however, data on the outcome of the use of these drugs in APOL1RRV individuals are not yet available. Since high glucose is the best known cytotoxin to podocytes, blood glucose control represents a key goal in diabetic APOL1RRV individuals. Since the majority of patients with type 2 diabetes mellitus do not lack insulin but rather are resistant to insulin action, agents improving metabolic profile (management of obesity with drugs, including GLP-1 agonists) in addition to drug therapy to control blood glucose are used. Because both hypertension and glucose elevation remain significant determinants of the progression of CKD, effective management of these parameters is of cardinal importance irrespective of the APOL1 risk allele genotype. APOL1 inhibition with emerging medications is still in clinical trials, predominantly in patients with chronic AMKD [67]. Identifiable adverse milieus, such as viral infection, remain a major player in the development of acute AMKDs; in these instances, treating the offending agent remains an effective therapeutic strategy.

## 17. Conclusions

We hypothesized that the ongoing miR193a-induced podocyte cytotoxicity was already at its peak in the diabetic milieu, which perhaps was not escalated further by second-hit-enhanced APOL1RRV expression. Similarly, the diabetic state was associated with a high level of podocyte RAS activation; the additional mild to moderate level of the RAS activation exacerbated by APOL1RRVs may be considered as unlikely to escalate the existing RAS levels any further. Regarding the BioMe repository analysis, we hypothesized that hypertension antedating the decline in GFR in individuals with APOL1RRVs was mediated by RAS activation in the form of systemic hypertension and glomerular injury simultaneously. However, the beginning of systemic hypertension was recorded at the onset of enhanced expression of APOL1RRVs. In contrast, glomerular dynamics and injury could not be measured at its onset, and the associated mild increase in GFR was masked by ongoing podocyte injury. Therefore, chronologically, hypertension antedated the decline in GFR.
